# From inflammatory to reconstitution: a longitudinal exploratory study of cytokine dynamics in HIV/TB co-infected patients

**DOI:** 10.1186/s12981-026-00926-w

**Published:** 2026-07-20

**Authors:** Zengjie Qian, Jianjian Li, Cuixian Yang, Mi Zhang, Xia Li, Junyi Zhao, Mei Yang, Hengli Liu, Youwang Lu, Hongli Fan

**Affiliations:** 1 Yunnan Infectious Diseases Hospital, Kunming, 650301 Yunnan China; 2 School of Public Health, Kunming Medical University, Kunming, 6500003 Yunnan China; 3 School of Public Health, Dali University, Dali, 671000 Yunnan China

**Keywords:** HIV/TB co-infection, Cytokine profile, Longitudinal monitoring, Immune reconstitution

## Abstract

**Background:**

Tuberculosis (TB) remains the most common opportunistic infection among people living with HIV (PLWH). Dysregulated cytokine responses during HIV/TB co-infection may contribute to immune dysfunction and disease progression. However, the longitudinal dynamics of cytokine profiles and their associations with clinical indicators during treatment remain incompletely understood.

**Methods:**

In this prospective exploratory longitudinal study, we enrolled 24 patients with HIV/TB co-infection, 24 patients with HIV monoinfection, and 24 healthy controls. Serum concentrations of seven cytokines, including IL-2, IL-4, IL-6, IL-10, IL-17, TNF-α, and IFN-γ, as well as routine clinical indicators, were measured at baseline and on days 7, 14, 21, 30, 60, 90, and 180 after treatment initiation. Longitudinal changes were analyzed using linear mixed-effects models, and associations between cytokines and clinical indicators were assessed using exploratory Spearman’s rank correlation analysis.

**Results:**

At baseline, patients with HIV/TB co-infection showed evidence of immune and inflammatory alterations, with higher NEUT, NEUT%, and AST levels and lower LYM, LYM%, and ALB levels than both comparison groups (FDR-adjusted *P* < 0.05). During follow-up, IL-6 and IL-2 peaked on days 7 and 60, respectively, whereas IFN-γ, TNF-α, and IL-17 peaked on day 21 and subsequently declined. IL-4 and IL-10 peaked on days 60 and 21, respectively, and decreased to levels comparable to those in healthy controls by days 180 and 90, respectively. Exploratory correlation analyses showed time-dependent changes in the associations between cytokines and clinical indicators. The correlations of TNF-α with NEUT and IFN-γ with CD4⁺ T cells and LYM shifted from negative to positive, whereas the correlation between IL-17 and CD8⁺ T cells shifted from positive to negative.

**Conclusion:**

Patients with HIV/TB co-infection showed stage-specific changes in cytokine profiles and clinical indicators during combined antiretroviral and anti-tuberculosis therapy. Cytokine levels and their associations with clinical indicators changed dynamically and gradually approached a relatively stable state over time. These findings suggest that immune and inflammatory status may evolve during treatment, although further studies are needed to validate these exploratory observations.

## Introduction

Tuberculosis (TB), caused by *Mycobacterium tuberculosis* (MTB), is the most common opportunistic infection among PLWH. The risk of developing active TB in PLWH is approximately 20-fold higher than that in the general population [1, 2]. According to the latest data from the World Health Organization (WHO), TB caused approximately 150,000 deaths among PLWH worldwide in 2024 and remains one of the leading causes of death in this population [3]. The synergistic interaction between HIV and MTB continues to pose a major challenge to global public health.

Cytokines are key regulators of immune function and play important roles in multiple aspects of HIV and MTB pathogenesis, including immune responses, inflammation, hematopoiesis, and cellular proliferation and differentiation [4]. HIV and MTB co-infection may disrupt cytokine homeostasis, leading to changes in the expression of both pro-inflammatory and anti-inflammatory cytokines. These alterations may reflect the host immune response to dual-pathogen infection and contribute to the distinct immunological profile observed in co-infected individuals [5].

Previous studies have shown that HIV infection leads to CD4⁺ T-cell depletion and impairs the anti-mycobacterial functions of innate immune cells, including macrophages and neutrophils, thereby increasing susceptibility to TB. Conversely, chronic inflammation associated with MTB infection may further promote HIV replication and immune dysfunction, contributing to a cycle of immune impairment and inflammatory activation [6, 7]. Key cytokines, such as IFN-γ, IL-4, and IL-10, have been associated with MTB persistence, disease progression, and clinical outcomes [8]. However, the longitudinal dynamics of these cytokines during treatment for HIV/TB co-infection, as well as their relationships with clinical indicators, remain incompletely understood.

The mechanisms underlying innate immune responses in HIV/TB co-infection and the effects of antiretroviral therapy on immune reconstitution remain incompletely understood. Previous studies have primarily focused on the immunopathological interactions between HIV and MTB and the associated immune dysregulation, whereas longitudinal evidence on changes in cytokine networks and their relationships with clinical indicators remains limited [5, 9–11]. In the present study, patients with HIV/TB co-infection were followed longitudinally at multiple time points, and their immune parameters were compared with those of patients with HIV monoinfection and healthy controls. We systematically evaluated dynamic changes in cytokine profiles and clinical indicators during treatment and explored their correlations over time. This study aimed to characterize stage-specific features of immune and inflammatory responses and to provide evidence that may inform the assessment of inflammatory status and future immunological investigations in this population.

## Methods

### Study population

Between May 2023 and June 2025, 30 patients with HIV/TB co-infection were initially enrolled. Patients with concomitant acute or chronic infections, including viral hepatitis, pneumonia, or cryptococcosis, and those with incomplete clinical data were excluded, resulting in 24 eligible patients in the HIV/TB group. In addition, 24 PLWH receiving antiretroviral therapy (ART) without other infectious diseases and 24 healthy individuals recruited from the same hospital during the same period were included as comparison groups. This study was reviewed and approved by the Ethics Committee of Yunnan Provincial Hospital of Infectious Diseases (Approval No. 2022028). Written informed consent was obtained from all participants prior to enrollment.

HIV/TB co-infection was diagnosed according to the Expert Consensus on the Diagnosis and Treatment of Tuberculosis in Patients with HIV/AIDS (2025 Edition) [12]. HIV infection was screened using enzyme-linked immunosorbent assay (ELISA) and confirmed by Western blot. Pulmonary TB was confirmed by molecular or microbiological testing: 15 cases were confirmed by Xpert MTB/RIF, and nine cases were confirmed by positive sputum or bronchoalveolar lavage fluid cultures.

All patients received standard combined antiretroviral and anti-tuberculosis therapy. The ART regimen included tenofovir disoproxil fumarate (TDF), raltegravir (RAL), and emtricitabine (FTC), whereas the anti-tuberculosis regimen consisted of isoniazid, rifampicin, ethambutol, and pyrazinamide (HRZE). Patients in the HIV/TB group were followed at baseline and on days 7, 14, 21, 30, 60, 90, and 180 after treatment initiation, during which physical examinations and laboratory assessments were performed.

All patients with HIV/TB co-infection were receiving ART at the time of pulmonary TB diagnosis. ART was continued without interruption after TB diagnosis, and anti-tuberculosis treatment was initiated immediately. At baseline, 16 patients had an HIV-1 viral load of < 200 copies/mL. By day 30, all patients had achieved or maintained viral suppression below this threshold, and no virological rebound was observed throughout follow-up.

### Clinical data and sample collection

Demographic characteristics, including sex, age, body mass index (BMI), and comorbidities, were collected from all participants. Clinical data, including chest CT findings and routine laboratory parameters, such as complete blood count, liver and kidney function tests, and T-lymphocyte subsets, were obtained from patients in the HIV/TB group.

At each follow-up visit, 5 mL of peripheral venous blood was collected into anticoagulant-free tubes and centrifuged at 3000 rpm for 15 min. Serum samples were separated and stored at − 80 °C until cytokine analysis.

### Serum cytokine concentration assay

Serum concentrations of IL-2, IL-4, IL-6, IL-10, IL-17, TNF-α, and IFN-γ were measured using a double-antibody sandwich flow cytometric fluorescence assay with a commercial cytokine detection kit (Hunan Weigong Biotechnology Co., Ltd., Hunan, China). The linear detection range for all analytes was 2.4–2500 pg/mL, with a correlation coefficient (r) ≥ 0.970. Intra-assay precision was assessed using samples with cytokine concentrations of 8 ± 5 pg/mL and 300 ± 100 pg/mL, and the coefficient of variation (CV) was ≤ 15% for all analytes.

The assay used seven fluorescence-coded magnetic microsphere populations, each coated with a cytokine-specific mouse anti-human monoclonal capture antibody. After incubation with serum samples, bound cytokines were detected using fluorescence-labeled mouse anti-human monoclonal detection antibodies, forming sandwich immune complexes. Fluorescence signals from individual microsphere populations were acquired by flow cytometry, and cytokine concentrations were calculated from analyte-specific standard curves.

The manufacturer-provided reference ranges for serum cytokines were as follows: IL-2 ≤ 7.2 pg/mL, IL-4 ≤ 7.7 pg/mL, IL-6 ≤ 9.1 pg/mL, IL-10 ≤ 7.5 pg/mL, IL-17 ≤ 8.8 pg/mL, IFN-γ ≤ 13.2 pg/mL, and TNF-α ≤ 14.8 pg/mL.

### Determination of absolute counts of peripheral blood T-lymphocyte subsets

A single-platform flow cytometric method was used to determine the percentages and absolute counts of CD3^+^, CD4^+^, and CD8^+^ T-lymphocyte subsets in peripheral blood samples. Analyses were performed using a BD FACSLyric flow cytometer (Becton, Dickinson and Company, BD). Fluorochrome-conjugated monoclonal antibodies against CD3, CD4, and CD8, as well as BD Trucount™ absolute counting tubes, were obtained from the same manufacturer.

All procedures were performed according to the manufacturer’s instructions. Lymphocytes were identified using a CD45/side scatter (SSC) gating strategy. CD3⁺ T cells within the lymphocyte gate were further classified into CD3⁺CD4⁺ and CD3⁺CD8⁺ subsets. Absolute counts (cells/µL) and percentages of each subset were automatically calculated based on the ratio of cellular events to fluorescent reference beads in the BD Trucount™ system.

### Statistical analysis

Statistical analysis was performed using SPSS 26.0 software, with graphical representations generated via OriginPro 2025 and Adobe Illustrator 2025.

Continuous variables were expressed as mean ± standard deviation or median with interquartile range (IQR), depending on data distribution. Categorical variables were presented as frequencies and percentages. Group comparisons were performed using the chi-square test or Fisher’s exact test for categorical variables, as appropriate. For continuous variables, one-way analysis of variance (ANOVA) was used for normally distributed data, whereas the Kruskal–Wallis test was applied for non-normally distributed data. Effect sizes were reported as standardized mean difference (SMD). All statistical tests were two-sided, and *P* < 0.05 was considered statistically significant.

Longitudinal changes in clinical and immunological parameters were analyzed using linear mixed-effects models, with time treated as a fixed effect and subject-specific variability included as a random effect. Regression coefficients (*β*) represented the average change in outcomes per unit increase in time. These models were used to characterize within-subject longitudinal trends over follow-up and were not adjusted for additional clinical covariates because of the relatively small sample size and the exploratory nature of the study.

To control for multiple comparisons in group comparisons and longitudinal analyses, the Benjamini–Hochberg false discovery rate (FDR) procedure was applied, with FDR < 0.05 considered statistically significant. Correlation analyses were exploratory in nature, and no additional adjustment for multiple comparisons was applied. Normality was assessed using the Shapiro–Wilk test, and most variables were found to be non-normally distributed (*P* < 0.05); therefore, Spearman’s rank correlation analysis was used. Missing data were handled using listwise deletion, with no imputation performed.

### Result

#### Demographic characteristics of the three study groups

A total of 72 participants were included in this study, comprising 24 individuals in each of the HIV/TB group, HIV monoinfected group, and healthy control group (NC). No statistically significant differences were observed among the three groups with respect to age, sex, or the prevalence of hypertension, chronic kidney disease, diabetes, or immune reconstitution inflammatory syndrome (IRIS) (all *P* > 0.05).

Significant differences in BMI were observed among the groups (*P* < 0.05). The BMI in the NC group (28.54 ± 4.08 kg/m²) was higher than that in both the HIV/TB group (19.12 ± 3.23 kg/m²) and the HIV monoinfected group (19.21 ± 0.08 kg/m²), whereas no significant difference was observed between the two HIV-positive groups (Table 1**)**.

During follow-up, no cases of voluntary treatment discontinuation or interruption were recorded. Among the 24 patients in the HIV/TB group, three were lost to follow-up due to transfer to another hospital. The remaining patients completed the scheduled follow-up, and no treatment failure or death occurred. Overall, the clinical condition improved compared with baseline.

**Table 1:** Baseline demographic and clinical characteristics of participants in the three study groups

Characteristics	HIV/TB group	HIV group	NC group	*P*
Age (years)	47.42 ± 11.68	46.58 ± 11.55	45.08 ± 11.89	0.801
BMI (kg/m²)	19.12 ± 3.23	19.21 ± 0.08	28.54 ± 4.08	< 0.05
Gender				0.850
Male	18(75.0%)	16(66.7%)	16(66.7%)	
Female	6(25.0%)	8(33.3%)	8(33.3%)	
Hypertension				0.797
Yes	3(12.5%)	5(20.8%)	5(20.8%)	
No	21(87.5%)	19(79.2%)	19(79.2%)	
Chronic kidney disease				0.485
Yes	2(8.3%)	4(16.3%)	1(4.2%)	
No	22(91.7%)	20(83.3%)	23(95.8%)	
Diabetes mellitus				0.324
Yes	0(0%)	2(8.3%)	0(0%)	
No	24(100.0%)	22(91.7%)	24(100.0%)	
IRIS				0.468
Yes	5(20.8)	8(33.3%)		
No	19(79.2)	16(66.7%)		

Data are presented as mean ± SD or n (%)

## Dynamic changes in routine laboratory parameters

**Fig. 1:**
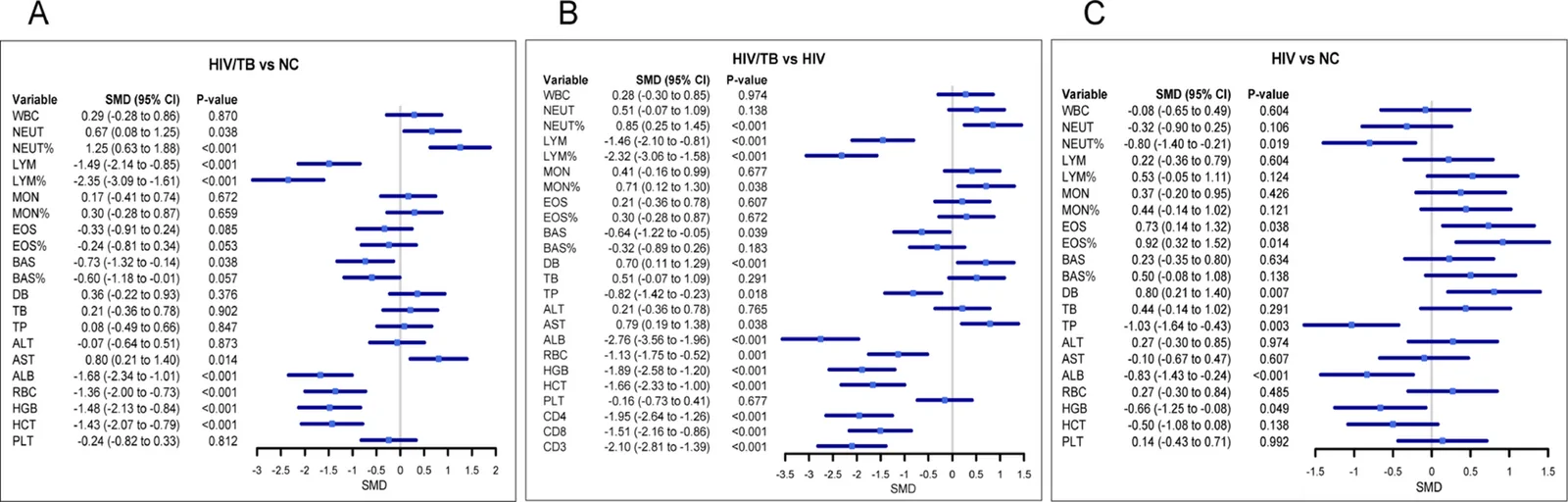
Comparison of standardized mean differences in baseline clinical measures across the three groups. **A** HIV/TB group vs. NC group; **B** HIV/TB group vs. HIV monoinfection group; **C** HIV monoinfection group vs. NC group. SMD, standardized mean difference; NC, healthy control group. Horizontal lines represent 95% confidence intervals

Before treatment, compared with the healthy control group, the HIV/TB group exhibited higher NEUT, NEUT%, and AST levels (SMD = 0.67, 1.25, and 0.80, respectively; all FDR-adjusted *P* < 0.05), whereas LYM, LYM%, BAS, ALB, RBC, HGB, and HCT levels were lower (all FDR-adjusted *P* < 0.05).(Figure 1A**).**

Compared with the HIV monoinfection group, the HIV/TB group showed higher direct bilirubin (DB) levels (SMD = 0.70; FDR-adjusted *P* < 0.05), whereas LYM%, CD3⁺ T cells, CD4⁺ T cells, and CD8⁺ T cells were lower (SMD = − 2.32, − 2.10, − 1.95, and − 1.51, respectively; all FDR-adjusted *P* < 0.05). In addition, NEUT% and AST levels were higher, whereas TP, ALB, RBC, HGB, and HCT levels were lower in the HIV/TB group than in the HIV monoinfection group (all FDR-adjusted *P* < 0.05). (Fig. 1B**).**

Compared with the healthy control group, the HIV monoinfection group exhibited higher EOS, EOS%, and DB levels (SMD = 0.73, 0.92, and 0.80, respectively; all FDR-adjusted *P* < 0.05), whereas NEUT%, TP, ALB, and HGB were lower (all FDR-adjusted *P* < 0.05 (Fig. 1C**).**

Linear mixed-effects model analysis showed that neutrophil count (NEUT) and NEUT% decreased significantly over time (*β* = −0.010 and − 0.091, respectively; both FDR-adjusted *P* < 0.05), with NEUT approaching the level observed in the healthy control group by day 21 of treatment. In contrast, LYM% and albumin (ALB) increased significantly during treatment (*β* = 0.065 and 0.026, respectively; both FDR-adjusted *P* < 0.05), with ALB approaching the level observed in the healthy control group after 90 days of treatment **(**Table 2**)**.

**Table 2:** Dynamic changes in laboratory parameters before and after treatment in patients with HIV/TB co-infection

Indicator	NC	HIV	Pre-T	DPT							
	(*n* = 24)	(*n* = 24)	(*n* = 24)	7d (*n* = 24)	14d (*n* = 24)	21d (*n* = 24)	30d (*n* = 24)	60d (*n* = 21)	90d (*n* = 21)	180d (*n* = 21)	β
WBC (×10^9^/L)	5.85± 1.80	5.97± 1.25	9.11± 15.56	5.45± 1.99	4.26± 2.02	4.42± 1.04	5.59± 2.93	4.58± 1.73	4.34± 1.46	4.66± 1.49	-0.016
NEUT (×10^9^/L)	3.16± 1.45	3.55± 0.85	4.35± 2.00^c^	4.06± 1.81	2.91± 1.38	3.37± 0.94	4.10± 2.44	2.81± 1.43	2.62± 1.23	2.79± 1.15	-0.010^d^
NEUT%	52.82± 9.24^a^	59.24± 6.20^b^	70.37± 17.14^c^	74.97± 10.63	67.20± 9.80	76.26± 6.02	71.91± 11.66	63.17± 15.51	60.54± 15.34	57.93± 8.83	-0.091^d^
LYM (×10^9^/L)	2.04± 0.66	1.91± 0.49^b^	0.95± 0.78^c^	0.72± 0.60	0.80± 0.54	0.61± 0.17	0.76± 0.69	1.00± 0.51	0.92± 0.58	1.24± 0.37	0.001
LYM%	36.29± 9.26	32.16± 5.79^b^	15.40± 8.21^c^	14.14± 6.80	19.08± 6.60	14.58± 3.41	14.14± 9.76	22.98± 11.87	23.12± 10.34	27.86± 8.11	0.065^d^
MON (×10^9^/L)	0.46± 0.16	0.40± 0.12	0.49± 0.29	0.46± 0.21	0.40± 0.23	0.38± 0.09	0.55± 0.25	0.45± 0.31	0.43± 0.26	0.44± 0.18	0.000
MON%	7.58± 2.09	6.78± 1.41^b^	8.30± 2.63	8.98± 3.21	10.33± 5.32	8.66± 4.19	11.13± 3.45	11.62± 6.10	10.59± 5.06	10.02± 4.43	0.016
EOS (×10^9^/L)	0.15± 0.10^a^	0.08± 0.06	0.11± 0.13	0.12± 0.13	0.11± 0.10	0.02± 0.03	0.11± 0.14	0.09± 0.05	0.21± 0.30	0.17± 0.18	0.001
EOS%	2.45± 1.42^a^	1.37± 0.81	1.95± 2.57	2.29± 2.37	2.67± 2.45	0.45± 0.41	2.25± 2.44	1.66± 1.20	5.08± 7.57	3.61± 3.42	0.015
BAS (×10^9^/L)	0.03± 0.02	0.03± 0.01^b^	0.02± 0.02^c^	0.02± 0.02	0.03± 0.03	0.01± 0.01	0.03± 0.03	0.02± 0.01	0.02± 0.01	0.03± 0.02	0.000
BAS%	0.56± 0.21	0.48± 0.12	0.40± 0.32	0.45± 0.31	0.72± 0.57	0.34± 0.36	0.46± 0.47	0.32± 0.19	0.46± 0.23	0.56± 0.37	0.001
DB (umol/L)	5.00± 3.68^a^	2.80± 0.98^b^	7.62± 9.49	5.46± 7.09	7.44± 9.33	3.08± 2.27	3.69± 1.94	4.53± 2.40	4.48± 2.17	7.47± 7.81	0.006
TB (umol/L)	11.89± 6.03	9.54± 4.41	13.73± 10.54	9.34± 8.99	11.31± 10.53	6.26± 4.20	6.88± 3.13	9.52± 4.69	10.52± 2.22	10.73± 4.58	-0.004
TP (umol/L)	71.15± 6.20^a^	76.70± 4.14^b^	71.73± 7.33	69.51± 8.58	71.24± 10.07	77.99± 7.54	70.38± 5.21	67.87± 7.34	72.83± 9.00	73.50± 7.15	0.007
ALT (U/L)	26.25± 17.43	22.42± 8.63	25.12± 15.70	14.21± 9.63	11.50± 6.47	10.00± 3.79	13.00± 6.35	18.81± 8.63	16.94± 8.12	14.11± 7.74	-0.044
AST (U/L)	22.42± 6.36	22.96± 4.34^b^	33.71± 18.47^c^	27.86± 15.94	26.21± 12.48	29.64± 6.20	22.46± 4.06	23.56± 5.47	25.28± 6.44	21.67± 9.64	-0.060
ALB (g/L)	44.80± 6.78^a^	49.14± 2.54^b^	31.84± 8.35^c^	29.09± 6.95	29.30± 7.18	31.47± 3.69	31.03± 4.15	30.91± 2.73	38.63± 6.08	37.76± 5.23	0.026^d^
RBC (×10^12^/L)	4.92± 0.47	4.77± 0.60^b^	3.76± 1.09^c^	3.61± 1.04	3.55± 1.12	3.17± 0.41	3.48± 0.66	3.47± 1.17	3.57± 1.08	3.93± 0.94	0.001
HGB (g/L)	148.54± 16.02^a^	160.04± 18.01^b^	111.62± 30.73^c^	107.29± 25.31	105.07± 28.20	88.45± 6.06	104.29± 21.57	105.31± 19.53	113.50± 32.37	125.22± 25.34	0.054
HCT%	43.86± 3.88	46.17± 5.12^b^	33.75± 9.04^c^	32.19± 7.62	32.06± 8.52	28.57± 1.47	31.80± 5.94	32.04± 5.61	34.05± 8.93	36.90± 6.43	0.011
PLT (×10^9^/L)	229.54± 96.64	218.62± 51.15	208.35± 73.23	255.43± 57.12	201.50± 90.00	192.64± 28.99	210.21± 92.12	228.56± 91.99	201.44± 77.63	186.11± 51.93	-0.169
CD4^+^ (cells/µl)		620.08± 260.67^b^	160.67± 198.44	171.00± 188.08	132.07± 145.22	94.36± 63.45	93.87± 85.41	202.25± 241.37	184.40± 109.35	226.20± 189.50	0.135
CD8^+^ (cells/µl)		839.42± 351.95^b^	383.71± 229.05	403.38± 238.99	385.00± 181.77	338.55± 177.66	278.13± 171.47	362.00± 226.33	446.60± 394.81	491.00± 259.62	0.673
CD3^+^ (cells/µl)		1477.25± 464.64^b^	564.00± 386.23	586.66± 393.42	536.68± 286.95	434.36± 190.56	362.67± 238.41	566.38± 357.41	633.20± 496.71	728.73± 351.34	0.879

Data are presented as mean ± SD or n (%)

ᵃSignificant difference between the NC group and the HIV monoinfection group (FDR-adjusted *P* < 0.05)

ᵇsignificant difference between the Pre-T group and the HIV monoinfection group (FDR-adjusted *P* < 0.05)

ᶜsignificant difference between the Pre-T group and the NC group (FDR-adjusted *P* < 0.05)

ᵈsignificant temporal trend identified using linear mixed-effects model analysis (FDR-adjusted *P* < 0.05). CD3⁺, CD4⁺, and CD8⁺ T-cell data were not available for the NC group

### Dynamic alterations in pro-inflammatory and anti-inflammatory cytokine profiles

**Fig. 2:**
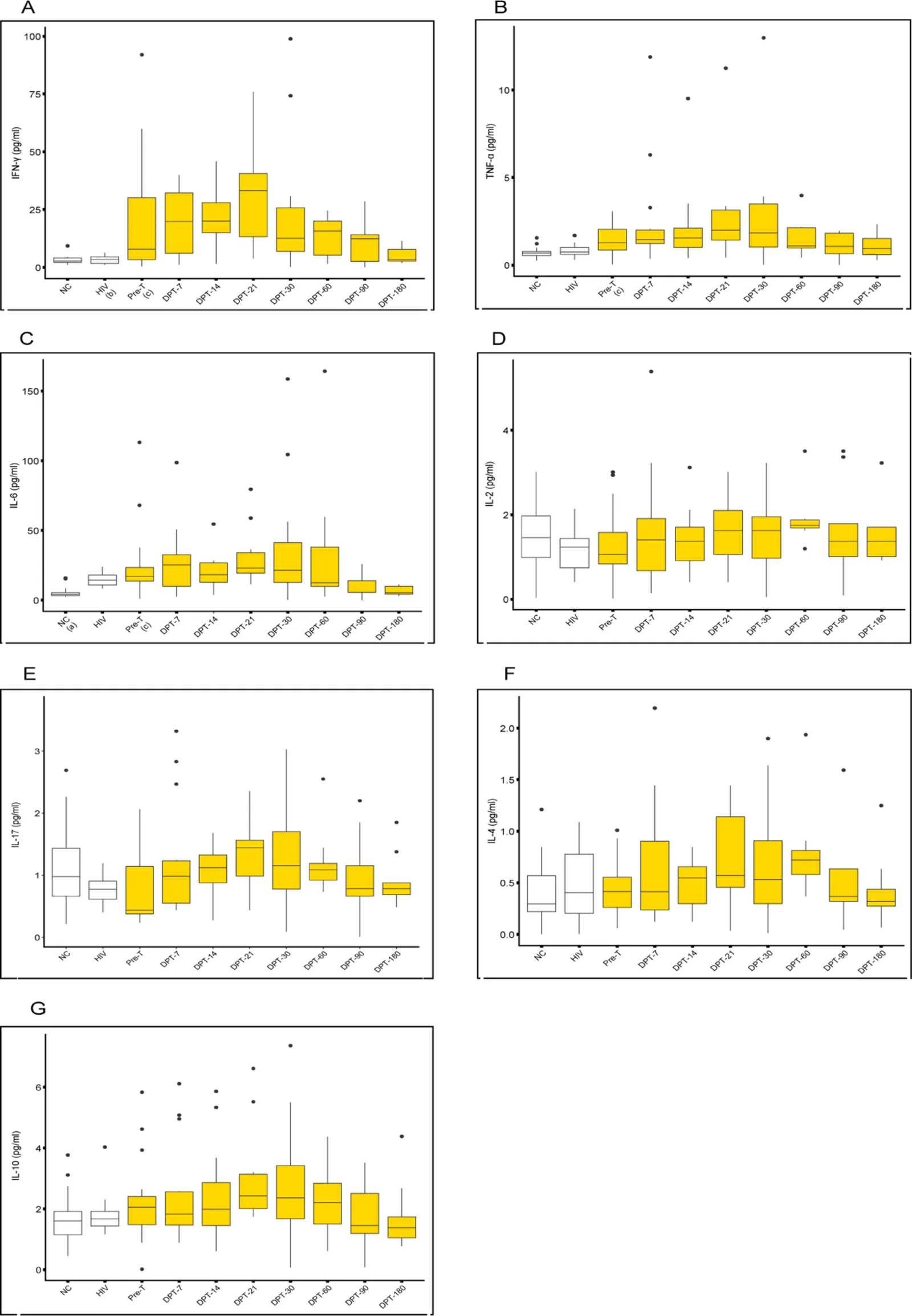
Dynamic changes in serum cytokine levels during treatment in patients with HIV/TB co-infection. Panels **A**–**E** show pro-inflammatory cytokines, including IFN-γ, TNF-α, IL-6, IL-2, and IL-17, and panels **F**–**G** show anti-inflammatory cytokines, including IL-4 and IL-10. NC, healthy control group; HIV, HIV monoinfection group; Pre-T, pretreatment; DPT-x, day x post-treatment. Letters indicate statistically significant differences among groups after FDR correction (FDR-adjusted *P* < 0.05): a, NC vs. HIV monoinfection; b, HIV monoinfection vs. Pre-T; c, NC vs. Pre-T

At baseline, most of pro-inflammatory cytokines were higher in the HIV/TB group than in the HIV monoinfection and NC groups, and cytokine trajectories showed a phased temporal pattern. Specifically, IFN-γ levels were higher in the HIV/TB group than in both comparison groups (FDR-adjusted *P* < 0.05). IFN-γ increased during early treatment, peaked on day 21, and subsequently declined, returning to the manufacturer-provided reference range by day 90. TNF-α levels were higher in the HIV/TB group than in the NC group at baseline (FDR-adjusted *P* < 0.05) and decreased progressively after peaking on day 21. IL-6 levels were higher in the HIV/TB group than in both the NC and HIV monoinfection groups at baseline (FDR-adjusted *P* < 0.05), peaked rapidly on day 7, and returned to baseline levels by day 60. No significant between-group differences were observed in IL-2 or IL-17 levels. IL-2 peaked on day 60 and approached the level observed in the NC group by day 90, whereas IL-17 decreased gradually after peaking on day 21 of treatment **(**Fig. 2A–E**)**.

No significant differences were observed in IL-4 or IL-10 levels among the groups (FDR-adjusted *P* > 0.05). IL-4 levels were similar to those in the HIV monoinfection group at baseline, peaked on day 60, and returned to levels comparable to those in the NC group by day 180. Baseline IL-10 levels tended to be higher in the HIV/TB group than in both the NC and HIV monoinfection groups, peaked on day 21, and returned to levels close to those in the NC group by day 90 **(**Fig. 2F–G**).**

### Correlation between cytokines and clinical indicators

TNF-α was negatively correlated with NEUT before treatment (*r* = − 0.50, *P* < 0.05). This correlation became positive on day 14 after treatment initiation (*r* = 0.22), and weakened on day 30 (*r* = 0.14). TNF-α showed little correlation with MON% before treatment (*r* = − 0.08); however, a strong negative correlation was observed on day 14 after treatment initiation (*r* = − 0.84, *P* < 0.05). The strength of this correlation gradually decreased on days 21 and 30 (*r* = − 0.54 and − 0.17, respectively) and became positive on day 180 (*r* = 0.58).

IFN-γ was negatively correlated with CD4⁺ T-cell counts before treatment (*r* = − 0.52, *P* < 0.05). This correlation became positive on day 14 after treatment initiation (*r* = 0.42), weakened on days 21 and 30 (*r* = 0.20 and − 0.09, respectively), and became negative again on day 180 (*r* = − 0.33). IFN-γ was also negatively correlated with LYM before treatment (*r* = − 0.50, *P* < 0.05). This correlation became positive on day 14 after treatment initiation (*r* = 0.14), strengthened on day 21 (*r* = 0.81, *P* < 0.05), and became negative again on day 30 (*r* = − 0.16).

**Fig. 3:**
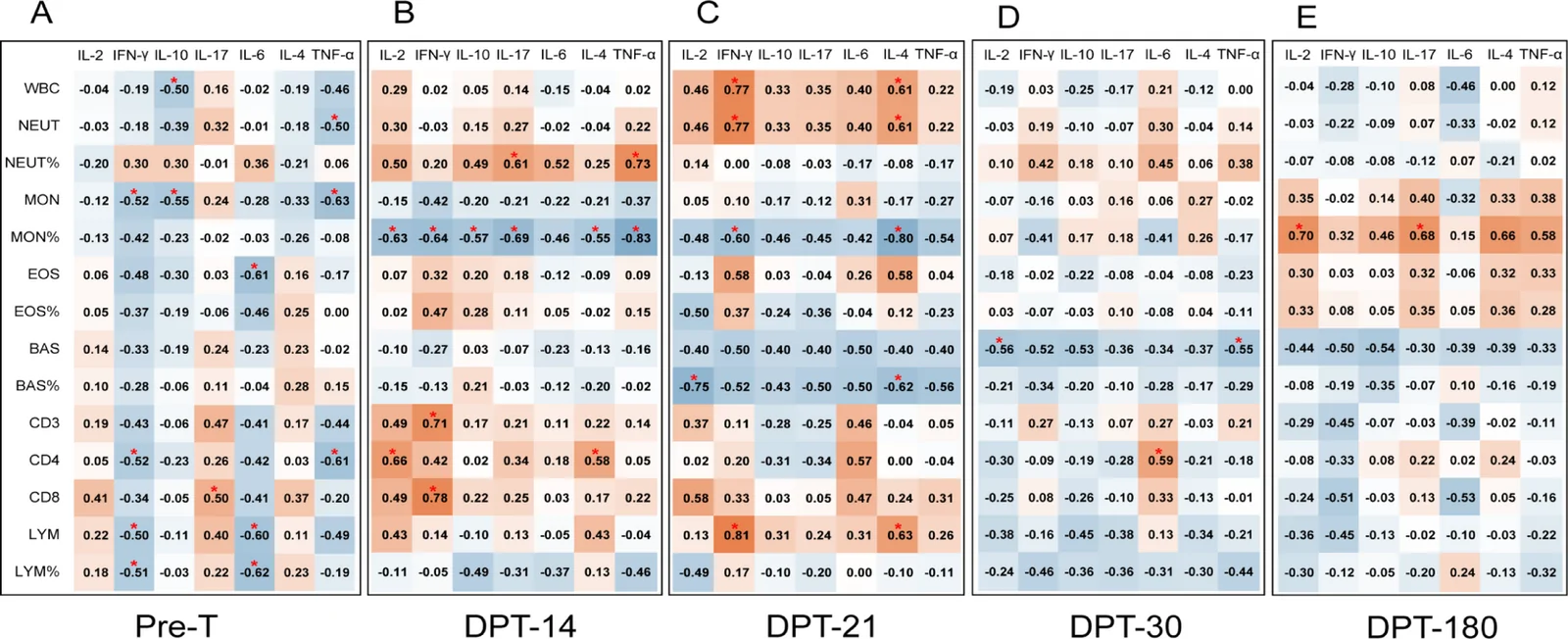
Correlation heatmap between clinical indexes and cytokines in patients with HIV/TB co-infection. Panels **A**–**E** show correlations between clinical indicators and cytokines at different treatment time points. Pre-T, pretreatment; DPT-x, day x post-treatment. *P < 0.05. Correlation analyses were exploratory, and no adjustment for multiple comparisons was applied

IL-17 was positively correlated with CD8⁺ T-cell counts before treatment (*r* = 0.50, *P* < 0.05). The strength of this correlation gradually decreased during treatment, became negative on day 30 (*r* = − 0.10), and became positive again on day 180 (*r* = 0.13). (Fig. 3A-E**).** These correlation analyses were exploratory, and no adjustment for multiple comparisons was applied.

## Discussion

In this study, patients with HIV/TB co-infection were followed longitudinally for 6 months at multiple time points to characterize dynamic changes in cytokine profiles and their associations with clinical indicators during combined antiretroviral and anti-tuberculosis therapy. Several inflammation-related cytokines were elevated during the early phase of treatment, which may reflect a state of immune activation during this period. As treatment progressed, cytokine levels and their associations with clinical indicators showed stage-specific changes and gradually approached a relatively stable state.

Before treatment, patients with HIV/TB co-infection showed evidence of immune dysfunction and multi-system involvement, characterized by adaptive immune suppression, innate immune activation, impaired liver function, and hematopoietic abnormalities. In the present study, CD4⁺, CD8⁺, and CD3⁺ T-cell counts, as well as LYM and LYM%, were reduced at baseline, suggesting impaired adaptive immune status associated with dual infection. This finding is consistent with that reported by Khayumbi et al. [13]. Previous studies have reported that MTB infection may be associated with enhanced HIV-specific T-cell exhaustion and persistent immune activation in HIV/TB co-infection, which may provide a possible background for T-cell dysfunction and reduced peripheral lymphocyte counts [7]. However, these mechanisms were not directly assessed in the present study. The reductions in EOS and EOS% may be associated with granulocyte redistribution during MTB infection. Although eosinophil infiltration into tuberculous granulomas has been reported, its relationship with peripheral eosinophil reduction remains uncertain [14]. Elevated AST and direct bilirubin levels, together with decreased TP and ALB levels, may indicate possible hepatic involvement and impaired synthetic function [15]. In addition, reductions in RBC, HGB, and HCT suggest anemia in patients with HIV/TB co-infection, which may be associated with chronic inflammation and increased metabolic demand [16].

Standardized combined antiretroviral and anti-tuberculosis therapy may be associated with gradual changes in immune-related and systemic clinical indicators in patients with HIV/TB co-infection [5]. In the present study, NEUT and NEUT% decreased during treatment, whereas LYM% and ALB increased. These changes may reflect a gradual reduction in inflammatory activity and partial improvement in immune-related and nutritional or hepatic synthetic status during follow-up [17]. Among these indicators, ALB showed a continuous upward trend, which is consistent with previous reports describing gradual recovery during treatment in patients with HIV/TB co-infection [18]. This change may be related to improved hepatic protein synthesis or overall clinical recovery [19].

The stage-specific changes in cytokine profiles may reflect dynamic immune and inflammatory responses in patients with HIV/TB co-infection. Elevated pro-inflammatory cytokines before treatment may be associated with host responses to dual-pathogen infection. Previous studies have shown that MTB infection is commonly associated with Th1-type immune responses, including IFN-γ and TNF-α signaling, which are involved in macrophage activation and granuloma formation [20–22]. The differences in peak timing among pro-inflammatory cytokines may indicate temporal heterogeneity in inflammatory responses during treatment. IL-6 peaked early, which may reflect an early inflammatory response during the initial treatment phase [22, 23]. IFN-γ, TNF-α, and IL-17 peaked on day 21, suggesting transient enhancement of inflammation-related cytokine activity during early follow-up. This pattern may be associated with immune reconstitution and persistent antigenic stimulation after the initiation of anti-tuberculosis therapy [6, 7, 21]. IL-2 showed a delayed peak on day 60, which may be related to gradual changes in T-cell-related immune activity during treatment [6, 20].

The dynamic changes in anti-inflammatory cytokines observed in this exploratory longitudinal study may reflect changes in the balance between inflammatory and regulatory immune responses during treatment. IL-4 peaked on day 60, which may suggest a delayed regulatory or anti-inflammatory response following the early inflammatory phase. This pattern may be associated with the gradual resolution of inflammation during treatment. IL-10 peaked on day 21. Previous studies have described IL-10 as an important immunoregulatory cytokine in TB infection, with potential roles in modulating macrophage activation and Th1-type cytokine responses [24]. In the present study, the relatively higher IL-10 level before treatment may reflect a compensatory regulatory response to inflammation, whereas its subsequent decline during treatment may be associated with gradual improvement in inflammatory status.

In addition, BMI was significantly higher in the healthy control group than in the HIV monoinfection and HIV/TB groups. Previous studies have reported that BMI is associated with systemic inflammatory status and cytokine expression levels [25, 26]. Therefore, BMI may have influenced the between-group comparisons of cytokine levels in the present study. This potential confounding effect should be considered when interpreting differences in cytokine profiles among the study groups. Meanwhile, a small number of patients developed IRIS during follow-up. IRIS has been associated with exaggerated inflammatory responses and increased cytokine release after ART initiation [27]. Therefore, IRIS may have influenced early changes in inflammatory cytokines and immune status during treatment.

Exploratory correlation analyses suggested that the relationships between certain cytokines and clinical indicators changed dynamically during treatment. The changing correlations of TNF-α with neutrophils and monocytes, as well as those of IFN-γ with CD4⁺ T cells and lymphocytes, may indicate that the associations between inflammatory cytokine activity and cellular immune indicators vary across treatment stages, showing a stage-specific pattern [1, 5]. In addition, the dynamic correlations between IFN-γ and CD4⁺ T cells, together with the directional changes in the association between IL-17 and CD8⁺ T cells, may suggest temporal variability in cytokine–T-cell relationships during treatment. These exploratory findings are broadly consistent with previous observations regarding T-cell differentiation and functional changes [6, 28].

This study has several limitations. First, from a statistical perspective, the sample size was relatively small, and FDR correction was not applied to the exploratory correlation analyses, which may increase the risk of type I error. Although linear mixed-effects models were used to characterize longitudinal changes over time, fully adjusted multivariable mixed-effects models incorporating additional clinical covariates were not performed because of the limited sample size. Therefore, we could not determine whether the observed longitudinal changes or cytokine–clinical indicator associations were independent of time-varying effects or potential clinical confounders. Missing data were handled using complete-case analysis, which may also introduce potential bias. Second, from a methodological perspective, only peripheral blood cytokine levels were measured, whereas the cellular sources and subcellular localization of these cytokines were not investigated. This limited our ability to characterize cytokine production across specific immune cell subsets. Third, from a clinical perspective, baseline BMI differences between groups, IRIS, viral load suppression status, baseline immune status, and individual variability in treatment response may have influenced cytokine dynamics and the interpretation of immune status. In addition, because ART had been initiated before anti-tuberculosis treatment and was continued throughout the study period, prior ART exposure may have affected baseline immune status and influenced the longitudinal trajectories of cytokine levels during subsequent anti-tuberculosis therapy. Future studies should include larger multicenter cohorts and incorporate single-cell sequencing, functional assays, and fully adjusted longitudinal modeling to better characterize immune responses at the cellular subset level and further explore the biological basis of the observed immunological changes.

In summary, this exploratory longitudinal study showed that patients with HIV/TB co-infection exhibited distinct stage-specific immune changes during combined antiretroviral and anti-tuberculosis therapy. As treatment progressed, inflammatory cytokine levels and their associations with clinical indicators changed dynamically and gradually approached a relatively stable state. These findings suggest a gradual attenuation of inflammatory activity during treatment, although further studies are needed to validate these observations and clarify the underlying biological mechanisms.

## Data Availability

Data are available from the corresponding author on reasonable request.

## Abbreviations

ALB: Albumin
ALT: Alanine aminotransferase
AST: Aspartate aminotransferase
ART: Antiretroviral therapy
BAS: Basophil count
BAS%: Basophil percentage
BMI: Body mass index
DB: Direct bilirubin
EOS: Eosinophil count
EOS%: Eosinophil percentage
ELISA: Enzyme-linked immunosorbent assay
HCT: Hematocrit
HGB: Hemoglobin
HIV: Human immunodeficiency virus
IFN-γ: Interferon gamma
IL: Interleukin
IRIS: Immune reconstitution inflammatory syndrome
LYM: Lymphocyte count
LYM%: Lymphocyte percentage
MON%: Monocyte percentage
MTB: Mycobacterium tuberculosis
NC: Healthy control group
NEUT%: Neutrophil percentage
PLWH: People living with HIV
PLT: Platelet count
RBC: Red blood cell count
SMD: Standardized mean difference
TB: Tuberculosis
TNF-α: Tumor necrosis factor alpha
TP: Total protein
WBC: White blood cell count

## References

[1] Tan Y, Guo W, Zhu Q, Song S, Xiang Y, Wu S, et al. Characterization of peripheral cytokine-secreting cells responses in HIV/TB co-infection. Front Cell Infect Microbiol. 2023;13:1162420. 10.3389/fcimb.2023.1162420 PMC10359493 37483385

[2] Organization WH, HIV. and tuberculosis. 2023 [Available from: https://www.who.int/westernpacific/health-topics/hiv-aids/hiv-and-tuberculosis

[3] Organization WH. Global tuberculosis report 2025. 2025 [Available from: https://www.who.int/publications/i/item/9789240116924

[4] Chauhan P, Nair A, Patidar A, Dandapat J, Sarkar A, Saha B. A primer on cytokines. Cytokine. 2021;145:155458. 10.1016/j.cyto.2021.155458 33581983

[5] Gunawan CK, Sumarpo A, Indrati AR. Immune Dysregulation in HIV-TB Co-Infection: Role of Cytokines and T Cell Biomarkers-A Narrative Review. Pathogens (Basel Switzerland). 2026;15(1). 10.3390/pathogens15010051 PMC12845183 41599035

[6] Esmail H, Riou C, Bruyn ED, Lai RP, Harley YXR, Meintjes G, et al. The Immune Response to Mycobacterium tuberculosis in HIV-1-Coinfected Persons. Annu Rev Immunol. 2018;36:603–38. 10.1146/annurev-immunol-042617-053420 29490165

[7] Sharan R, Bucşan AN, Ganatra S, Paiardini M, Mohan M, Mehra S, et al. Chronic Immune Activation in TB/HIV Co-infection. Trends Microbiol. 2020;28(8):619–32. 10.1016/j.tim.2020.03.015 PMC7390597 32417227

[8] Auld SC, Staitieh BS. HIV and the tuberculosis set point: how HIV impairs alveolar macrophage responses to tuberculosis and sets the stage for progressive disease. Retrovirology. 2020;17(1):32. 10.1186/s12977-020-00540-2 PMC7509826 32967690

[9] Nosik M, Belikova MG, Ryzhov K, Avdoshina D, Sobkin A, Zverev V et al. Unique Profile of Proinflammatory Cytokines in Plasma of Drug-Naïve Individuals with Advanced HIV/TB Co-Infection. Viruses. 2023;15(6). 10.3390/v15061330 PMC10303992 37376629

[10] Walker NF, Meintjes G, Wilkinson RJ. HIV-1 and the immune response to TB. Future Virol. 2013;8(1):57–80. 10.2217/fvl.12.123 PMC3644063 23653664

[11] Ahmed A, Rakshit S, Vyakarnam A. HIV-TB co-infection: mechanisms that drive reactivation of Mycobacterium tuberculosis in HIV infection. Oral Dis. 2016;22(Suppl 1):53–60. 10.1111/odi.12390 27109273

[12] yinzhong S, Hongzhou L. Expert consensus on the diagnosis and treatment of HIV/AIDS patients co-infection with Tuberculosis(2025 edition). Chin J AIDS STD. 2025;31(11):1278–86.

[13] Khayumbi J, Sasser LE, McLaughlin TA, Muchiri B, Ongalo J, Tonui J, et al. Active Tuberculosis Is Associated with Depletion of HIV-Specific CD4 and CD8 T Cells in People with HIV. AIDS Res Hum Retroviruses. 2024;40(7):417–27. 10.1089/aid.2023.0088 PMC11295841 38366732

[14] Bohrer AC, Castro E, Hu Z, Queiroz ATL, Tocheny CE, Assmann M et al. Eosinophils are part of the granulocyte response in tuberculosis and promote host resistance in mice. J Exp Med. 2021;218(10). 10.1084/jem.20210469 PMC8348215 34347010

[15] Naidoo K, Hassan-Moosa R, Mlotshwa P, Yende-Zuma N, Govender D, Padayatchi N, et al. High Rates of Drug-induced Liver Injury in People Living With HIV Coinfected With Tuberculosis (TB) Irrespective of Antiretroviral Therapy Timing During Antituberculosis Treatment: Results From the Starting Antiretroviral Therapy at Three Points in TB Trial. Clin Infect diseases: official publication Infect Dis Soc Am. 2020;70(12):2675–82. 10.1093/cid/ciz732 PMC7931836 31622456

[16] O’Mahony D, Mabunda SA, Mntonintshi M, Iruedo J, Kaswa R, Blanco-Blanco E et al. Causes of Moderate and Severe Anaemia in a High-HIV and TB-Prevalent Adult Population in the Eastern Cape Province, South Africa. Int J Environ Res Public Health. 2023;20(4). 10.3390/ijerph20043584 PMC9966846 36834279

[17] Peluso MJ, Colby DJ, Pinyakorn S, Ubolyam S, Intasan J, Trichavaroj R, et al. Liver function test abnormalities in a longitudinal cohort of Thai individuals treated since acute HIV infection. J Int AIDS Soc. 2020;23(1):e25444. 10.1002/jia2.25444 PMC6968973 31953919

[18] Bisaso KR, Owen JS, Ojara FW, Namuwenge PM, Mugisha A, Mbuagbaw L, et al. Characterizing plasma albumin concentration changes in TB/HIV patients on anti retroviral and anti -tuberculosis therapy. silico Pharmacol. 2014;2(1):3. 10.1186/s40203-014-0003-9 PMC4173069 25264502

[19] Strauss KE, Phoswa WN, Hanser S, Mokgalaboni K. HIV Infection and Antiretroviral Therapy Impair Liver Function in People Living with HIV: Systematic Review and Meta-Analysis. Pharmaceuticals (Basel Switzerland). 2025;18(7). 10.3390/ph18070955 PMC12300453 40732246

[20] Hosseinian K, Gerami A, Bral M, Venketaraman V. Mycobacterium tuberculosis-Human Immunodeficiency Virus Infection and the Role of T Cells in Protection. Vaccines (Basel). 2024;12(7). 10.3390/vaccines12070730 PMC11281535 39066368

[21] Lyu J, Narum DE, Baldwin SL, Larsen SE, Bai X, Griffith DE, et al. Understanding the development of tuberculous granulomas: insights into host protection and pathogenesis, a review in humans and animals. Front Immunol. 2024;15:1427559. 10.3389/fimmu.2024.1427559 PMC11663721 39717773

[22] Li Q, Wang C, Gou J, Kitanovski S, Tang X, Cai Y, et al. Deciphering lung granulomas in HIV & TB co-infection: unveiling macrophages aggregation with IL6R/STAT3 activation. Emerg microbes infections. 2024;13(1):2366359. 10.1080/22221751.2024.2366359 PMC11188963 38855910

[23] Ritter K, Rousseau J, Hölscher C. The Role of gp130 Cytokines in Tuberculosis. Cells. 2020;9(12). 10.3390/cells9122695 PMC7765486 33334075

[24] Xu H, Jia Y, Li Y, Wei C, Wang W, Guo R, et al. IL-10 Dampens the Th1 and Tc Activation through Modulating DC Functions in BCG Vaccination. Mediat Inflamm. 2019;2019:8616154. 10.1155/2019/8616154 PMC6594250 31281230

[25] Kumar NP, Nancy AP, Moideen K, Menon PA, Banurekha VV, Nair D, et al. Low body mass index is associated with diminished plasma cytokines and chemokines in both active and latent tuberculosis. Front Nutr. 2023;10:1194682. 10.3389/fnut.2023.1194682 PMC10265642 37324745

[26] Mave V, Erlandson KM, Gupte N, Balagopal A, Asmuth DM, Campbell TB, et al. Inflammation and Change in Body Weight With Antiretroviral Therapy Initiation in a Multinational Cohort of HIV-Infected Adults. J Infect Dis. 2016;214(1):65–72. 10.1093/infdis/jiw096 PMC4907416 26962236

[27] Lai RP, Meintjes G, Wilkinson RJ. HIV-1 tuberculosis-associated immune reconstitution inflammatory syndrome. Semin Immunopathol. 2016;38(2):185–98. 10.1007/s00281-015-0532-2 PMC4779131 26423994

[28] Zhu JT, Helper Cell. Differentiation, Heterogeneity, and Plasticity. Cold Spring Harb Perspect Biol. 2018;10(10). 10.1101/cshperspect.a030338 PMC6169815 28847903

